# Binding of galectin-1 to breast cancer cells MCF7 induces apoptosis and inhibition of proliferation in vitro in a 2D- and 3D- cell culture model

**DOI:** 10.1186/s12885-016-2915-8

**Published:** 2016-11-08

**Authors:** Pamina Geiger, Barbara Mayer, Irmi Wiest, Sandra Schulze, Udo Jeschke, Tobias Weissenbacher

**Affiliations:** 1 Department of Obstetrics and Gynecology, LMU Munich-Innenstadt, Maistrasse 11, 80337 München, Germany; 2 Department of General, Visceral and Transplantation Surgery, Hospital of the LMU Munich, Marchioninistr 15, 81377 Munich, Germany

**Keywords:** Galectin 1, Thomsen-Friedenreich, MCF7, Spheroid, Proliferation, Apoptosis

## Abstract

**Background:**

Galectin-1 (gal-1) belongs to the family of β-galactoside-binding proteins which primarily recognizes the Galβ1-4GlcNAc sequences of oligosaccharides associated with several cell surface glycoconjugates. The lectin recognizes correspondent glycoepitopes on human breast cancer cells. Galectin-1 is expressed both in normal and malignant tissues. Lymphatic organs naturally possessing high rates of apoptotic cells, express high levels of Galectin-1. Furthermore galectin-1 can initiate T cell apoptosis. Binding of galectin-1 to trophoblast tumor cells presenting the oncofetal Thomsen-Friedenreich (TF) carbohydrate antigen inhibits tumor cell proliferation. In this study we examined the impact galectin-1 has in vitro on cell proliferation, apoptotic potential and metabolic activity of MCF-7 and T-47D breast cancer cells in dependence to their expression of the Thomsen-Friedenreich (TF) tumor antigen.

**Methods:**

For proliferation and apoptosis assays cells were grown in presence of 10, 30 and 60 μg gal-1/ml medium. Cell proliferation was determined by a BrdU uptake ELISA.

Detection of apoptotic cells was done by M30 cyto death staining, in situ nick translation and by a nucleosome ELISA method. Furthermore we studied the impact galectin-1 has on the metabolic activity of MCF-7 and T-47D cells in a homotypic three-dimensional spheroid cell culture model mimicking a micro tumour environment.

**Results:**

Gal-1 inhibited proliferation of MCF-7 cells (strong expression of the TF epitope) but did not significantly change proliferation of T-47D cells (weak expression of the TF epitope). The incubation of MCF-7 cells with gal-1 raised number of apoptotic cells significantly. Treating the spheroids with 30 μg/ml galectin-1 in addition to standard chemotherapeutic regimes (FEC, TAC) resulted in further suppression of the metabolic activity in MCF-7 cells whereas T-47D cells were not affected.

**Conclusions:**

Our results demonstrate that galectin-1 can inhibit proliferation und metabolic cell activity and induce apoptosis in breast tumor cell lines with high expression levels of the Thomsen-Friedenreich (TF) antigen in monolayer and spheroid cell culture models.

## Background

Galectins belong to the family of lectins and are defined by specifically binding β-galactosides and by a conserved sequence motif of amino acids in the carbohydrate recognition domain (CRD). The first family member to be described, Galectin-1 (gal-1), is a homodimeric protein with a single carbohydrate recognition domain of 134 amino acids [1]. It has been identified to be expressed in lymphoid organs such as the thymus and lymph nodes, in activated macrophages and T cells. Furthermore its expression is balancing immune tolerance [2]. LacNAc is the basic ligand recognized by gal-1, but it also shows increased avidity to multiple Galβ1-4GlcNAc sequences presented on branched N-linked or on repeating LacNAc-residues on N- and O-linked glycans. Having a single CRD, gal-1 associates non-covalently under physiological conditions to form a homodimer and such becomes functionally bivalent. The bivalent nature entails glycan-mediated cross-linking of cell surface receptors believed to be essential in inducing signaling events [3, 4]. Extracellularly, by binding its glycan ligands, gal-1, exerts various biological effects in different tissues and on cells, including cell adhesion [5, 6], metastasis [7], cell growth regulation [8, 9], immunosuppression [10] and apoptosis [3].

Treatment of breast cancer tumor cells with galectin-1 leads to reduced cell binding to laminin and plasma or placental fibronectin [11]. Increased binding potential for galectin-1 in breast cancer cells seems to correlate with a positive lymph node status and with tumor size and stage, whereas the presence of galectin-1 was identified as a factor that correlates with a lack of metastatic lesions in lymph nodes. These results indicate quantitative cell-type-dependent requirements of galectin ligand presentation during the metastatic cascade [11].

Gal-1 expression is also found in the placenta. The placenta plays a key role in balancing local immuntolerance which is essential for the mother to accept the embryo during pregnancy. This complex process of tolerance allowing the foetal survival is controlled at the embryo-maternal interface by factors deriving as well from decidualized endometrium as from the trophoblast itself. Trophoblasts display various strategies to evade the destructive attack of the maternal immune response including expression of non-classical MHC class I antigens and of complement regulatory proteins [12, 13]. Chorioncarcinoma cell lines were evaluated as an experimental model of trophoblast-derived immunoregulation [14]. We found a strong expression of the Thomsen-Friedenreich (TF) tumour antigen in the choriocarcinoma cell line BeWo [15, 16].

The TF antigen (galactose-β1-3 N-acetylgalactosamine; Galβ1-3GalNAcα1) is a tumor-associated disaccharide which is occluded by covering structures and inaccessible to the immune system on the cell surface in most healthy tissues. It is however exposed and immunoreactive on most human carcinomas and T-cell lymphomas [17]. Galectin-1 binding to BeWo trophoblast tumor cells presenting the TF antigen inhibits tumor cell proliferation [16]. Large amounts of TF tumor antigen have as well been detected on the outer surface membranes of human breast carcinomas [17, 18].

The TF antigen and galectins have also already been implicated in tumour cell adhesion and tissue invasion. Gal-1 and gal-3 appear to participate both in the homotypic aggregation of human breast carcinoma cells MDA-MB-435 and their adhesion to the endothelium. This adhesion seemed to be mediated involving TF antigen, as it could be inhibited by a TF-antigen specific peptide [19].

In a former study we showed that gal-1 shows apoptotic potential in the human breast cancer line MCF-7 in combination with additional stress stimuli like hyperthermia or the removal of CO_2_ and FCS for 20 h [20].

In this article we describe that the binding of gal-1 on human breast cancer cells can induce inhibition of proliferation and apoptosis in dependence of their expression of the TF antigen.

When examining basic biological tumor cell functions in vitro, conventional monolayer cultures can only act as a very limited cancer model when it comes to sustaining the characteristics of the original tumor in vitro. Three dimensional spheroid cultures of cancer cells may reflect properties of tumors better than those traditional monolayer cultures, since they come closer to the in vivo situation regarding cell differentiation, proliferation, and cell environment, i.e., cell-cell contacts and different growth areas [21–23]. In this article we also describe that in a homotypic spheroid model as well binding of gal-1 on human breast cancer cells can reduce metabolic cell activity in dependence of their expression of the TF antigen.

## Methods

### Breast cancer cell lines and galectin-1 treatment

For this study we used MCF-7 and T-47D human breast cancer cell lines obtained from ATCC. Cells were grown in DMEM (Biochrom, Germany) supplemented with 10 % v/v foetal calf serum (PAA, Germany) and 2 mM L-glutamin (Sigma-Aldrich, Munich, Germany), without antibiotics and antimycotics. For proliferation assays and apoptosis assays cells were grown in the presence of 10, 30 and 60 μg galectin-1 (Sigma-Aldrich) per ml serum + 10 % FCS for 48 h. Untreated cells were used as controls.

### Immunocytochemistry

Each cell line was investigated for TF antigen expression by immunocytochemistry. Cells were grown on three-well multitest slides (Roth, Karlsruhe, Germany) to subconfluency, then dried, wrapped and stored at -80 °C. After thawing, cells were briefly fixed with formalin (Merck, Darmstadt, Germany; 5 % in PBS (Biochrom), 5 min). The primary anti-TF antibody (Table 1) was diluted to 2 μg/ml with PBS and incubated with the slides overnight at 4 °C. After washing this was followed by incubation with the biotinylated secondary antibody from the Vectastain® Elite ABC Mouse IgG Kit (Vector Laboratories, Peterborough, UK) diluted 1:200 for 30 min. Furthermore we used the Vectastain® Elite ABC Kit for visualization according to the instructions of the manufacturer. The slides were finally embedded in mounting buffer and examined with a Zeiss (Jena, Germany) Axiophot photomicroscope. Images were aquired with a digital camera system (Axiocam, Zeiss).

**Table 1:** Antibodies used for the study

Antigen	Antibody	Isotype	Concentration/Dilution	Source
TF	NM-TF1	Mouse IgM	2 μg/ml	Glycotope
M30	ALX-804-590-T200	Mouse IgG	1:15	Alexis

### BrdU cell proliferation assay

Cell proliferation was analyzed with a 5-bromo-2′-deoxy-uridine (BrdU) labelling and detection kit (Roche Diagnostics GmbH, Mannheim, Germany) according to the manufacturer’s instructions. In 96-well tissue culture plates, cells (1 x 10^5^ in 0.1 ml cell culture medium) were grown for 72 h in the absence (controls) and presence of 10, 30 and 60 μg/ml gal-1. For labelling cells were incubated with BrdU for 3 h, then fixed and subsequently BrdU incorporation into the cellular DNA was measured by an ELISA technique. Cellular proliferation is expressed as percentage compared to the control. At least 8 replicates were performed with each cell line.

### M30 cytoDEATH apoptosis assay

Caspase activity is one of the earliest apoptosis markers. The M30 cytodeath assay detects caspase-cleaved Cytokeratin 18 in epithelial cells. Culture slides with MCF-7 cells grown in the presence of galectin-1 as described were treated according to the manufactures protocol (Alexis Biochemicals). Slides were washed in PBS and then fixed in ice-cold pure methanol at -20 °C for 30 min. After being washed twice with PBS they were incubated with M30 CytoDEATH Fluorescein antibody (Table 1) for 30 min at 15–25 °C and then washed again twice before immunocytochemical evaluation. 10 replicates were performed.

### *In situ* nick-translation (ISNT) apoptosis assay

The *in situ* nick-translation technique (ISNT) was used to staining DNA fragmentation and apoptotic bodies on cell culture slides [20]. Slides were incubated with proteinase K (20 μg/ml, Qiagen, Germany) for 15 min at room temperature. After rinsing with distilled water the endogenous peroxidase was quenched with 0.3 % hydrogen peroxide for 10 min. Being rinsed once more, the slideswere then equilibrated in nick buffer (Tris, MgCl_2_, ß-Mercaptoethanol, 20 mg/ml BSA, distilled water) at room temperature for 10 min. By incubating the slides with dNTPs and biotinylated 7-dATP (Gibco, USA) diluted in nick buffer for 65 min at 37 °C, the *in situ* nick-translation was performed. Terminating buffer (0.3 mol/L sodium chloride and 0.03 mol/L sodium citrate) was used to rinse the chamber slides at room temperature for 15 min. After having washed the slides in PBS, they were incubated with extravidin–peroxidase (Sigma, Germany) at room temperature for 30 min. AEC-substrate (Dako, Denmark) was used for colour development. Afterwards the slides were counterstained with haemalaun, then washed and mounted. The specificity of ISNT reactivity was confirmed by human epidermis and lymph node sections. 10 replicates were performed. Negative controls were performed by incubation in nick buffer without dNTPs and biotinylated 7-dATP.

### Immunocytochemical evaluation of apoptosis assays

For the evaluation of early apoptosis by M30 cytoDEATH staining and late apoptosis (*in situ* nick-translation) the intensity and distribution of the immunocytochemical staining reaction was evaluated using a semi-quantitative method (IRS-score) as previously described [24]. The rate of apoptosis for M30 cytoDEATH and *in situ* nick translation was determined by counting 1500 cells per chamberslide.

### Cell death detection ELISA

Apoptosis was also detected using a quantitative three-step photometric enzyme immunoassay. The Cell Death Detection ELISA^plus^ kit (Roche Diagnostics GmbH, Mannheim, Germany) detects cytoplasmic histone-associated DNA fragments (mono- and oligonucleosomes) in vitro after induced cell death. This assay uses monoclonal mouse antibodies directed against histones and DNA in a quantitative sandwich enzyme immunoassay. Specific mono- and oligonucleosomes in the cytoplasmic fraction of cell lysates can thus be detected. At first the anti-histone antibody was fixed adsorptively on the wall of the microplate where non-specific binding sites were saturated and hence blocked. Second the nucleosomes in the sample were bound to the immobilized anti-histone antibody via their histone component. Third, the DNA part of the nucleosome reacted with the anti-DNA-peroxidase. After washing unbound samples and reagents, the amount of peroxidase ligated in the immunocomplex was determined colorimetrically using ABTS as substrate. Results are presented in Units; Unit Conversion: 1 mU = 1 x 10-3 OD (1 mU = 0.001 OD). A total of 8 replicates were performed.

### Spheroid culture

3D cell culture was performed using a modified liquid overlay technique as described previously [25]. Briefly, monolayer cultures of the breast cancer cell lines MCF-7 and T-47D were allowed to reach a minimal confluency of 90 % for spheroid culture. The viability and the cell number of the cell suspensions used for spheroid culture were assessed. Only cell suspensions with a viability of at least 90 % were used for spheroid culture. For spheroid formation 5 × 10^4^ vital cells were seeded in 50 μl cell culture medium per 96-well and cultured for 48 h at 37 °C in a humidified atmosphere containing 5 % CO2. Using this approach, a single homotypic spheroid was obtained in each well.

### Cancer therapy and cell viability ATP-assay

After 48 h of spheroid formation, chemotherapeutic agents, namely fluorouracil combined with epirubicin and cyclophosphamide (FEC) and docetaxel combined with doxorubicin and cyclophosphamide (TAC) were administered to the spheroids in clinically relevant combinations at the peak plasma concentrations as described previously [26]. Galectin-1 was applied in a concentration of 30 μg/ml. Medium (untreated) and solvent controls were included in each experiment. Solvents used to control the effect of the drugs were 0.2%H2O plus 0.26 % NaCl for FEC therapy, 0.01 % H2O plus 0.21 % NaCl for TAC treatment and 0.15 % phosphate buffered saline (PBS) for galectin-1. Each treatment and control was performed in six replicates. The drugs were allowed to take effect for a total of 48 h. Chemotherapeutics were obtained from the pharmacy of the University Hospital LMU (Munich, Germany). Treatment efficacy was assessed using an ATP assay (CellTiter-Glo® Luminescence Cell Viability Assay, G8461, Promega, Germany) to quantify cell survival in vitro. Mean cell survival was expressed as percent of residual metabolic activity relative to the solvent controls.

### Statistical analysis

IBM SPSS Statistics for Windows, Version 22.0. ((IBM, Ehningen,Germany) was used for collection, processing, and statistical data analysis. The non-parametrical Wilcoxon test for comparison of the means was used for statistical analysis. P-values <0.05 were considered statistically significant. For statistical analysis of the results obtained in the spheroid model, the student’s *t*-test was performed for comparison of two samples. For comparisons of more than two samples, analysis of variance (ANOVA) with post-hoc Sidak correction was done.

## Results

### Expression of TF antigen in breast cancer cell lines

**Fig. 1:**
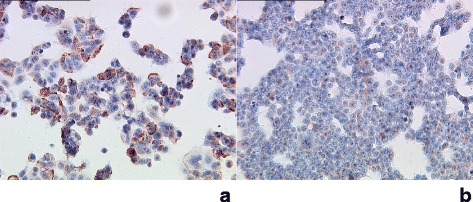
Strong expression of TF in MCF-7 cells (**a**). T47-D cells (**b**) showed only weak expression of TF. All magnification 10x lens

Expression of the Thomsen-Friedenreich (TF) antigen as a target for gal-1 binding was investigated in human breast cancer cells of the cell lines MCF-7 and T-47D by immunocytochemistry. Staining results are presented in Fig. 1. MCF-7 cells showed strong expression (Fig. 1a) whereas T-47D showed only weak expression of the TF epitope (Fig. 1b). All magnification 10x lens.

### Cell proliferation assay

**Fig. 2:**
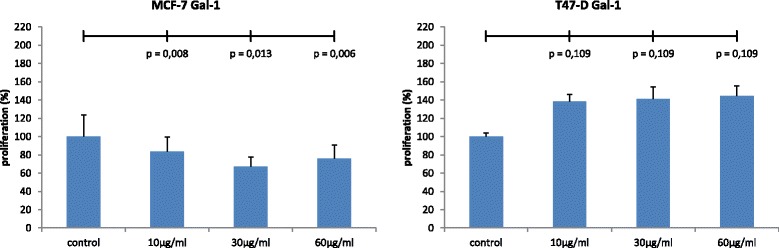
Gal-1 inhibits proliferation of MCF-7 cells in a concentration-dependent manner (*n =* 8). Significant decreases in cell proliferation were induced by treatment of the cultures with 10 μg/ml (*p =* 0.008), 30 μg/ml (*p =* 0.013) μg/ml and 60 μg/ml gal-1 (*p =* 0.006), respectively (**a**). Gal-1 did not significantly stimulate proliferation of T47D cells at concentrations of 10 μg/ml, 30 μg/ml, and 60 μg/ml (*p =* 0.109) (**b**)

As demonstrated in Fig. 2a, gal-1 inhibits proliferation of MCF-7 cells in a concentration-dependent manner. The addition of gal-1 at 10 μg/ml, 30 μg/ml, and 60 μg/ml reduced cellular 5-bromo-2^′^-deoxy-uridine (BrdU)-uptake significantly to 83.8 % (*p* = 0.008), 67.4 % (*p* = 0.013), and to 76.2 % (*p* = 0.006) respectively, compared to non-treated control cultures (100 %). Gal-1 did not significantly stimulate proliferation of T-47D cells at concentrations of 10 μg/ml, 30 μg/ml, and 60 μg/ml (*p* = 0.109) (Fig. 2b).

### Evaluation of apoptosis by M30 cytoDEATH

**Fig. 3:**
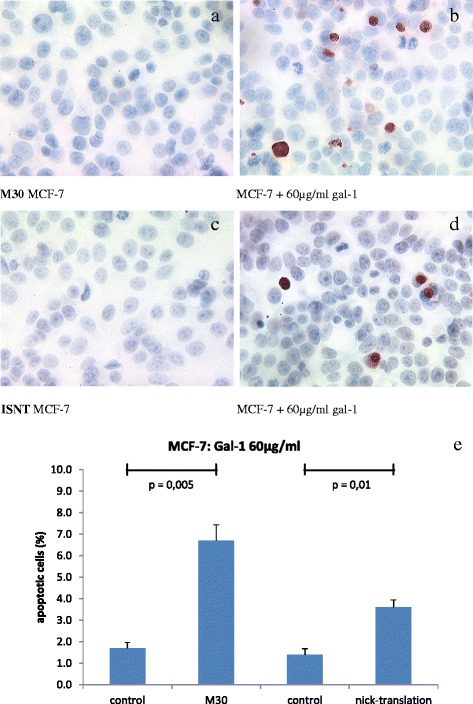
M30 staining in untreated for MCF-7 cells had a mean of 1.7 % (**a**). In cells treated with 60 μg/ml gal-1 for 48 h the rate of very early apoptosis is elevated to up to 6.7 % (*p =* 0.005, **b**). The normal rate of apoptosis in MCF-7 breast cancer cells had a mean of 1.4 % detected by in situ nick translation (ISNT, **c**). The incubation with 60 μg/ml gal-1 for 48 h significantly enhanced apoptosis in MCF7-cells to a maximum of 3.6 % (*p =* 0.01, **d**). Results of M30 and ISNT staining are summarized (**e**) (*n =* **10**)

The rate of very early apoptosis detected by M30 staining in untreated for MCF-7 cells had a mean of 1.7 % (Fig. 3a, e) evaluated by a semi-quantitative method. In cells treated with 60 μg/ml gal-1 for 48 h the rate of very early apoptosis is elevated to up to 6.7 % for MCF-7 cells (*p* = 0.005, Fig. 3b, e).

### Evaluation of apoptosis by in situ nick-translation (ISNT)

The normal rate of apoptosis in MCF-7 breast cancer cells had a mean of 1.4 % detected by ISTN (Fig. 3c, e). The viability of the cells manifested itself in a regular growth and a good range of mitosis. The exposure with 60 μg/ml gal-1 for 48 h significantly increased apoptosis in MCF7-cells up to 3.6 % (*p* = 0.01, Fig. 3d, e).

### Evaluation of apoptosis by cell death detection ELISA

**Fig. 4:**
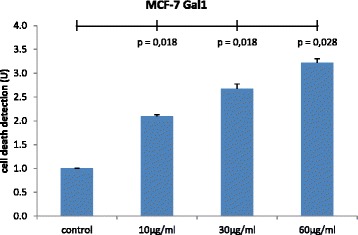
The incubation of MCF-7 cells with 10, 30 and 60 μg/ml gal-1 enhanced DNA fragmentation and nucleosoma formation, reaching statistically significance for 10, 30 and 60 μg/ml gal-1 incubation (*p =* 0,018; *p =* 0,018; *p =* 0.028) (*n =* 8)

DNA fragmentation was quantified by examining the cytoplasmic histone-associated DNA fragments (mononucleosomes and oligonucleosomes). The incubation of MCF-7 cells with 10, 30 and 60 μg/ml gal-1 enhancing apoptosis to a maximum of 2.1, 2.7 and 3.2 U, respectively (Fig. 4), reaching statistically significance for 10 μg/ml (*p* = 0.018), 30 μg/ml (*p* = 0.018) and 60 μg/ml (*p =* 0.028) gal-1 incubation.

### Evaluation of metabolic activity in the spheroid model

**Fig. 5:**
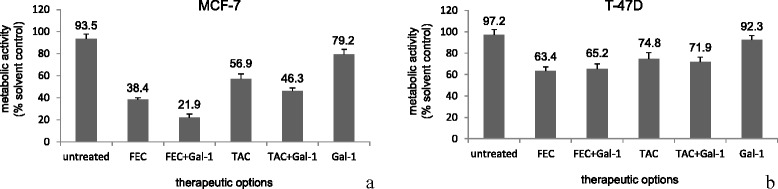
Measurement of the metabolic activity of human breast cancer cells in spheroid culture in the ATP assay. Significant decrease of metabolic activity could be reached by incubation of MCF-7 cells with 30 μg/ml gal-1 compared to untreated cells (*p =* 0.027). In combination with 1xPPC FEC (*p =* 0.016) or 1xPPC TAC (*p =* 0.031) 30 μg/ml gal-1 further significantly reduced the metabolic activity compared to FEC or TAC alone (**a**). In T-47D cells treatment with 30 μg/ml gal-1 could not significantly reduce the metabolic activity compared to untreated cells. Neither did the addition of 30 μg/ml gal-1 to 1xPPC FEC or 1xPPC TAC significantly alter the rate of metabolic activity compared to FEC or TAC alone (**b**)

Homotypic spheroids were prepared from human breast cancer cells lines and treated with various agents. Metabolic activity after treatment was measured using the ATP assay (Fig. 5). Treatment of MCF-7 cells with 30 μg/ml gal-1 decreased the metabolic activity to 79.16 % of solvent control compared to untreated cells (93.5 %) (*p =* 0.027). In combination with 1xPPC FEC 30 μg/ml gal-1 further reduced the metabolic activity to 21.9 % of solvent control compared to only 38.4 % FEC alone (*p =* 0.016). The same could be shown for combination of 30 μg/ml gal-1 with 1xPPC TAC which led to a reduction to 46.3 % of solvent control compared to 56.9 % TAC alone (*p =* 0.031) (Fig. 5a).

In T-47D cells the treatment of homotypic spheroids with 30 μg/ml gal-1 could not significantly reduce the metabolic activity to 92.3 % of solvent control compared to untreated cells (97.2 %). Also the addition of 30 μg/ml gal-1 to 1xPPC FEC did not significantly alter the rate of metabolic activity (65.2 % of solvent control compared to 63.4 % for FEC alone). Neither could the addition of 30 μg/ml gal-1 to 1xPPC TAC induce a significant effect (71.9 % of solvent control compared to 74.8 % for TAC alone) (Fig. 5b).

## Discussion

Within this study we could show that MCF-7 breast cancer cells show a strong expression of the TF antigen or epitope. Ligation of galectin-1 induced inhibition of proliferation as well as metabolic cell activity and onset of apoptosis.

The Thomsen-Friedenreich (TF) antigen [27] has been known as a tumour-associated antigen for a long time [28]. Masked and covered for example by covalently linked carbohydrates or physically seperated from the immune system, TF tumor antigen is present in most tissues on the surfaces of healthy cells. In its unsubstituted immunoreactive form it can frequently be found in cancer and precancerous conditions and in many of these cases, the increased TF occurrence correlates with the formation of metastasis and cancer progression [29]. The immunoreactive TF antigen also is expressed by fetal epithelia [30], can be found on transferrin isolated from human amniotic fluid [31] and is expressed by the syncytiotrophoblast and extravillous trophoblast [15]. The absence of TF in an immunoreactive form in non-carcinomatous postfetal tissues, its presence during an early fetal phase and its frequent occurance in carcinomas suggest that TF is a stage-specific oncofetal carbohydrate antigen.

In epithelial cells, it is mainly associated with mucin-1 (MUC1), a protein belonging to a family of highly glycosylated proteins lining the apical surface of many glandular epithelial cells. On tumour cells MUC1 is posttranslational modified leading to an exposure of the TF epitope by incomplete *O*-glycosylation. In several tumour entities, like colon [32], lung [33] or gastric cancer [34], or in cancer of the cervix uteri [35, 36], a correlation between TF expression and negative prognosis could be identified. Yet, in other tumour locations, like in breast cancer, its prognostic impact is indeterminate. On the one hand high TF expression predicted improved survival [37], then again another study identified a correlation between high tumour stage and TF expression [38].

In a former study we could demonstrate that in breast cancer patients TF is expressed on disseminated tumor cells in bone marrow (DTC-BM) as well [39]. As there is little knowledge which of the primary tumours’ factors correlates with haematogenous dissemination, we have also investigated the expression of TF antigen of breast cancer tissues from patients with known BM status at the time of first diagnosis. Patients with TF-positive tumours had a favourable prognosis [40]. This contrasts to studies on gastrointestinal tumours [41]. We hypothesised that at least three factors, dissemination routes, TF-mediated metastasis formation and the immunogenicity of TF, together determine the different prognostic impact of TF expression in different tumour locations [40].

Results obtained within this study demonstrate that gal-1 only shows apoptotic potential in TF-expressing breast tumor cell lines together with inhibition of proliferation. Breast cancer cells which expressed lower levels of TF showed no onset of apoptosis upon incubation with gal-1. In a preliminary study of our group apoptosis could be induced by gal-1 and additional stimuli like hyperthermia or long term removal of CO_2_ and FCS [20]. At a concentration of 60 μg/ml incubation of the cells with gal-1 for 48 h, as done in the present study, no further stimulus was needed to significantly increase apoptosis in the 2-D model.

Apart from studying the tumor biological effects gal-1 induces in a traditional monolayer culture model, we also tested them in a homotypic spheroid model. This model can come closer to mimicking the assembly of a tumor since spheroids consist of proliferating and viable but post-mitotic cell populations as well as cells and compact structures, often in the spheroid core, which may contain necrotic or apoptotic cells [23].

In the homotypic spheroid cell culture model incubation of MCF-7 cells with 30 μg/ml gal-1 for 48 h led to an significantly decreased level of metabolic activity especially when combined with standard chemotherapeutic regimes (FEC, TAC), whereas T-47D cells did not respond to gal-1 treatment. Therefore we hypothesize that gal-1 acts via TF on MCF-7 breast cancer cells.

## Conclusion

Downregulation of tumour cell proliferation and onset of apoptosis by ligation of the TF epitope in breast cancer patients could be a first step of new therapeutic options. For applications in earlier development phases, homotypic tumor cell line spheroid models are the preferable choice to determining the impact of treatment on cancer cells separated out of a cell mixture in a complex tumor. But in vivo tumor tissue is a complex micro environmental structure, not only consisting of the organ specific tumor cells, but also of various types of stromal cells as well as the extra-cellular matrix and different soluble factors. Therefore predicting biological response to drug treatment in a 2D- or homotypic 3D model cannot perfectly reflect in vivo conditions. In a study in which spheroids were either generated homotypic from colon cancer tumor cell lines, or tumor cell lines co-cultured with stromal cells or spheroids directly prepared from colon cancer tissues, the three spheroid models reacted differently to the treatment with clinically relevant cancer combination therapies [25]. Therefore to evaluate the putative relevance galectin-1 and the ligation of the TF epitope could have in breast cancer treatment regimes, testing in heterotypic spheroid models could provide further information.

## Abbreviations

ATP: Adenosine tri-phosphate
CRD: Carbohydrate recognition domain
FEC: Fluorouracil (F), epirubicin (E), cyclophosphamide (C)
Gal-1: Galectin-1
TAC: Docetaxel (T), doxorubicin (A), cyclophosphamide (C)
TF: Thomsen-Friedenreich epitope
